# Development of morphologically restored and deproteinized* Juglans regia* pollen-derived sporopollenin exine capsules: A multi-analytical verification study

**DOI:** 10.1038/s41598-026-53033-1

**Published:** 2026-05-22

**Authors:** Funda Ersoy Atalay, Seher Guler, Harun Kaya, Senol Alan

**Affiliations:** 1 The Faculty of Science and Arts, Department of Physics, Inonu University, Malatya, Türkiye Turkey; 2 The Faculty of Engineering and Natural Sciences, Malatya Turgut Ozal University, Malatya, Türkiye Turkey; 3 Faculty of Science, Department of Biology, Zonguldak Bulent Ecevit University, Zonguldak, Türkiye Turkey

**Keywords:** Sporopollenin exine capsules, *Juglans regia* pollen, deproteinized microcarriers, Solid-state ¹³C-CP/MAS NMR, PEG-4000 stabilization, SDS-PAGE deproteinization, Biochemistry, Biological techniques, Biotechnology, Plant sciences

## Abstract

Sporopollenin exine capsules (SECs) are attractive natural microcarriers, but allergenic protein residues remain a critical limitation for biomedical applications. In this study, the development of morphologically restored and deproteinized JSECs from *Juglans regia* pollen was verified. High-purity capsules were isolated via sequential chemical treatments, followed by a PEG-4000-assisted stabilization process to resolve structural collapse observed during acidolysis. Protein elimination and morphological recovery were validated using SDS-PAGE, solid-state ¹³C CP/MAS NMR, and BET analysis. The absence of detectable protein bands in SDS-PAGE, coupled with attenuation of peptide-associated signals in NMR and FT-IR, indicates that allergenic protein content was reduced below the detection limit of these methods. Morphological analyses revealed that PEG treatment effectively restored the spherical capsule structure, increasing surface area to 13.56 m^2^/g with a ~ 25% yield. Statistical analysis (*n* = 150) confirmed an 83.3% morphological recovery rate, demonstrating that PEG-4000 effectively prevents structural collapse while maintaining intact, spherical capsules. These results demonstrate that *Juglans*-derived capsules can be rendered with high purity while preserving structural integrity, highlighting their potential as sustainable natural carriers for future biomedical applications.

**Supplementary Information:**

The online version contains supplementary material available at https://doi.org/10.1038/s41598-026-53033-1.

## Introduction

Pollen grains are highly resilient natural microstructures that protect the male gametes of plants against environmental stress factors, with this exceptional stability primarily originating from the outer wall layer known as the exine. The exine is composed of sporopollenin, a highly cross-linked and chemically robust biopolymer. The complex three-dimensional network of sporopollenin, formed by aliphatic and aromatic structural units, provides remarkable resistance against harsh acidic and alkaline environments, elevated temperatures, and biological degradation while maintaining structural integrity^1,2^. These unique characteristics render sporopollenin exine capsules (SECs) particularly attractive as natural, long-lasting, and chemically stable microcarrier systems.

In recent years, sporopollenin-based capsules have attracted increasing interest across a wide range of applications, including drug delivery, controlled release systems, functional additives, and adsorption technologies. Compared to synthetic microcapsules, pollen- or spore-derived sporopollenin structures offer significant advantages such as species-specific uniform size distribution, predefined morphology, and derivation from renewable biological resources^3,4^. Moreover, these structures can be converted into hollow capsules through chemical extraction without the need for complex polymerization or templating processes, thereby enhancing both production reproducibility and sustainability^5,6^.

Sporopollenin is widely recognized as one of the most resilient biopolymers in the plant kingdom, known for its extraordinary resistance to harsh acidic/alkaline environments and biological degradation^1^. In the literature, the use of these hollow exine capsules (SECs) derived from pollen and spores as a natural model for drug delivery systems was first detailed by Diego-Taboada et al.^3^. Subsequently, Mundargi et al.^4^ developed optimized protocols for sporopollenin extraction from various plant species, demonstrating the drug-loading capacities and the selective absorption of single-stranded DNA (ssDNA). Today, sporopollenin research has evolved towards the production of bio-mimetic templates and clinically safe microcarriers from sustainable biomass sources, guided by the recent work of Aylanc et al.^5,6^ and De Mori et al.^7^. Furthermore, engineered SECs have recently demonstrated significant potential in specialized clinical areas, such as colon-targeted delivery and antioxidant therapy for inflammatory conditions^8,9^. However, the potential for preclinical development of such sophisticated delivery systems largely depends on the substantial reduction of allergenic protein residues below immunogenic thresholds, which remains a primary concern for the safety and biocompatibility of pollen-derived materials^10^. These technological advancements have positioned sporopollenin-based capsules as a strategic alternative across a broad biomedical spectrum, including not only controlled drug release but also the development of bio-functional materials.

Despite these advantages, the majority of studies on sporopollenin-based carrier systems have predominantly focused on a limited number of pollen types, particularly commercially available species such as *Lycopodium clavatum*, which exhibit relatively homogeneous size distributions^3^. This narrow focus introduces important limitations in terms of cost, accessibility, and long-term sustainability, while the structural diversity and functional potential of sporopollenin derived from alternative plant species remain largely underexplored. In recent years, research efforts have shifted towards evaluating local biomass resources, such as *Pistacia* and *Juglans* species, to identify sustainable and high-performance microcarrier templates^11^. In this context, *Juglans regia* (walnut) pollen emerges as a promising yet largely unexplored biological resource for the production of sporopollenin exine capsules. In addition to its potential in biomedical applications, the structural robustness of *Juglans* sporopollenin has also been demonstrated in high-performance energy storage systems^12^. In regions where walnut cultivation is widespread, large quantities of pollen are released into the environment during the pollination period and subsequently become an underutilized biomass after fertilization. This characteristic positions walnut pollen as a low-cost, locally accessible, and sustainable raw material. However, for its safe integration into biomedical and pharmaceutical applications, the efficient removal of pollen-derived allergenic proteins and a detailed assessment of capsule integrity following chemical processing are essential.

One of the primary limitations in the application of pollen-derived sporopollenin capsules is the presence of residual allergenic proteins and glycoproteins. The majority of pollen allergens are proteinaceous in nature and are known to trigger immune responses at the molecular level, posing potential risks for biomedical use^13^. Although various chemical protocols for sporopollenin extraction have been reported, protein removal efficiency has often been evaluated indirectly using techniques such as FTIR spectroscopy or elemental analysis. Direct biochemical and molecular-level validation of allergen removal remains limited in the literature^14,15^, leading to uncertainty regarding the reliability of sporopollenin-based systems described as “allergenic-free.”

Over the last five years, sporopollenin exine capsules (SECs) have transitioned from basic templates to versatile, high-performance biomaterials. Recent research has demonstrated their significant efficiency in environmental remediation, specifically for the sequestration of heavy metal ions^16^ and the removal of organic dyes from industrial effluents^17^. Concurrently, the biomedical applications of SECs have expanded to include mucoadhesive platforms for enhanced drug delivery^18^ and controlled drug delivery systems^19,20^. Furthermore, emerging technologies such as colon-targeted antioxidant therapy^8^ and bio-hybrid nanozyme systems^9^ highlight the growing clinical importance of these natural micro-carriers. These global advancements reinforce the necessity for the rigorous deproteinization and morphological restoration protocols established in this work. The present study distinguishes itself from existing literature by adopting a comprehensive approach that simultaneously verifies allergenic protein removal from sporopollenin exine capsules derived from *Juglans regia* pollen at both biochemical and molecular levels. Protein elimination was directly confirmed using SDS–PAGE, while FTIR and solid-state NMR analyses provided molecular-level evidence of successful purification. The results further demonstrate that the capsule structure preserves its morphological and structural integrity following chemical treatment. Overall, this study offers a systematic and innovative framework that elucidates the relationship between allergen safety and functional applicability of sporopollenin-based natural microcapsules.

## Materials and methods

### Materials

*Juglans regia* pollens were obtained from male catkins collected during the spring season from walnut trees located on the Inonu University campus (Malatya, Turkey). The collected catkins were shade-dried, and the pollens were allowed to release spontaneously without any mechanical intervention.

Prior to chemical treatment, the pollens were dehumidified and fractionated using an automatic sieving system (Retsch GmbH, AS 200 basic) compliant with ASTM E11 standards to remove foreign particles. The sieving process was conducted using mesh sizes of 212, 106, 75, 45, 38 and 25 μm (Mesh No. 70–500). Based on previous SEM analyses indicating that the characteristic dimensions of *Juglans regia* pollens exceed 25 μm, the fraction retained on the 25 μm (Mesh No. 500) sieve was utilized for the experimental studies. This pre-treatment process enhanced sample homogeneity and ensured the removal of unwanted dust and solid debris.

Acetone, orthophosphoric acid (H₃PO₄, 85%), potassium hydroxide (KOH, pellets), and polyethylene glycol (PEG 4000, M̅ₙ ≈ 4000) were of analytical grade and obtained from commercial sources. All solutions were prepared using deionized water with a resistivity of 18 MΩ·cm.

### Extraction and purification of *Juglans regia* sporopollenin exine capsules (JSECs)

**Fig. 1:**
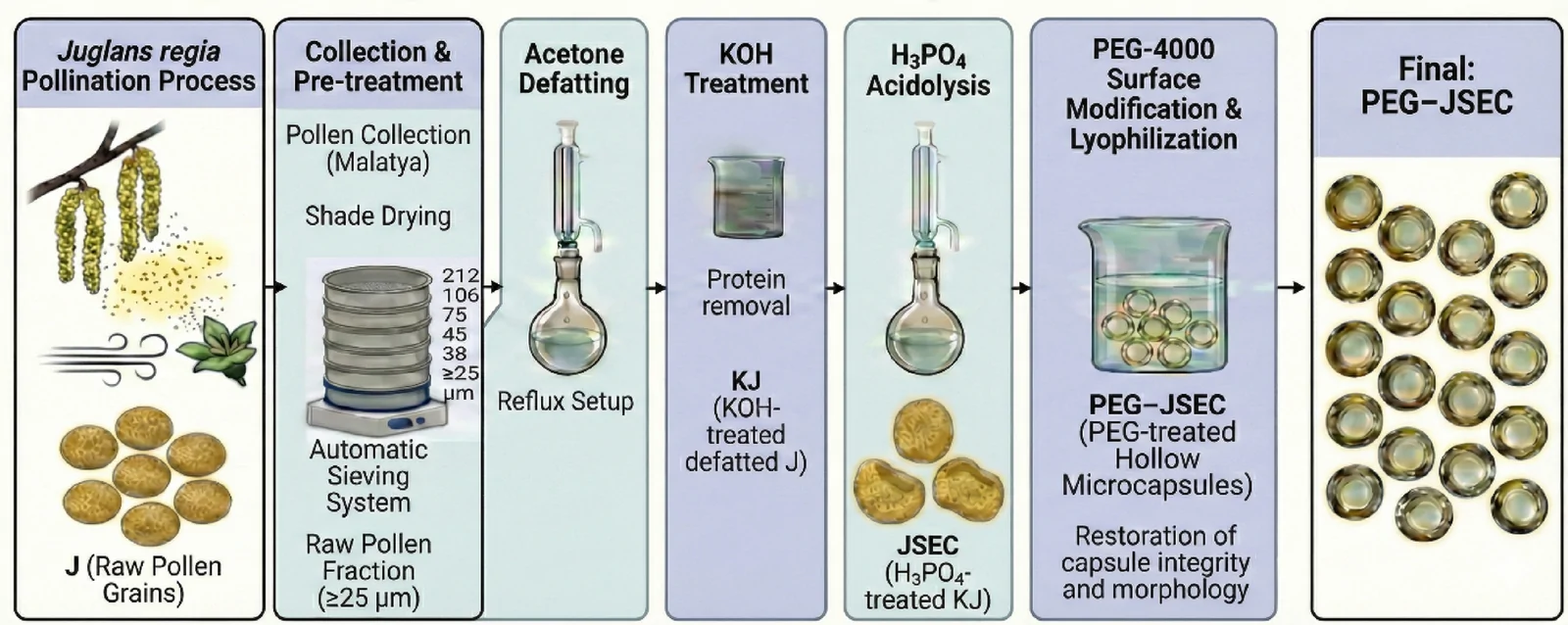
Schematic representation of the stepwise conversion of raw *Juglans regia* pollen grains (J) into hollow JSEC microcapsules via acetone defatting followed by sequential KOH and H₃PO₄ treatments, and PEG-4000-assisted stabilization to obtain the final PEG–JSEC.

Successive chemical purification steps were implemented to remove allergenic protein and lipid components from *Juglans regia* pollen and to obtain structurally stable, hollow sporopollenin exine capsules (JSECs). The outline of the methodology is illustrated as a diagram in Fig. 1. In this study, untreated isolated pollen grains are denoted as J, alkaline-treated pollen as KJ, and the sporopollenin exine capsules obtained through the sequential application of alkaline and acidic treatments as JSEC.

In preliminary trials, various combinations of the sequence of alkaline (KOH) and acidic (H₃PO₄) treatments were evaluated. It was observed that when the phosphoric acid treatment was applied in the early stages, the rapid removal of the internal structure led to the depletion of the capsule’s internal volume, resulting in significant structural collapse. Therefore, to better preserve the integrity of the capsules in the current study, the alkaline treatment was performed prior to phosphoric acid hydrolysis, and the acidic treatment was carried out as the final step. While the followed method partially resembles protocols reported in the literature, it has been specifically optimized considering the unique structural characteristics of *Juglans regia* pollen.

Figure 1 Schematic representation of the stepwise conversion of raw *Juglans regia* pollen grains (J) into hollow JSEC microcapsules via acetone defatting followed by sequential KOH and H₃PO₄ treatments, and PEG-4000-assisted stabilization to obtain the final PEG–JSEC.

#### Acetone defatting

Pollen (20 g), having undergone pre-screening processes, was transferred into a 500 mL round-bottom flask, and 200 mL of acetone was added. The mixture was kept under reflux conditions in an Elma TL-H-5 MF2 ultrasonic bath (45 kHz, 60% power) at 60 °C for 45 min, followed by continued reflux under magnetic stirring for an additional 30 min at the same temperature. Upon completion, the suspension was cooled to room temperature, the acetone was removed via vacuum filtration, and the solid phase was dried in an oven at 70 °C. This step was performed to eliminate lipidic components and acetone-soluble hydrophobic extractives.

#### Alkaline treatment

The defatted and dried pollen samples were transferred into a 500 mL flat-bottom flask containing 200 mL of 6% (w/v) KOH solution. The suspension was treated under reflux at 60 °C for 30 min with magnetic stirring. After the process, the sample was cooled, filtered via vacuum filtration, and washed at least three times with deionized water to remove alkaline residues. The resulting samples were designated as KJ. This step was applied to denature and remove proteins and to break down alkali-sensitive polysaccharide structures.

#### Phosphoric acid treatment (Acidolysis)

The wet pollen obtained after the KOH treatment was transferred directly (without drying) into a 500 mL flat-bottom flask, and 100 mL of 85% (w/w) H₃PO₄ was added. The mixture was treated under reflux conditions in an ultrasonic bath (45 kHz, 60% power) at 70 °C for 45 min, followed by continued acidic treatment under magnetic stirring for 30 min at the same temperature. An 85% H₃PO₄ concentration, which is relatively harsher than standard protocols, was specifically selected to ensure the complete hydrolysis of the dense intine layer and the rigorous elimination of internal allergenic proteins characteristic of the robust *Juglans regia* pollen wall. During this process, the cytoplasmic content and inner wall components were largely removed, ensuring the evacuation of the capsule’s internal volume. Once the reaction was complete, the sample was cooled to room temperature and centrifuged at 9,000 rpm and 4 °C for 15 min. The supernatant was discarded, and the solid phase was washed at least three times with pure water. The sporopollenin exine capsules obtained from this step were designated as JSEC.

#### PEG surface modification and lyophilization

The sporopollenin exine capsules obtained after the acidic and alkaline treatments were treated with polyethylene glycol (PEG 4000) to reduce structural collapse resulting from the significant evacuation of the internal volume and to improve capsule integrity and surface stability. For this purpose, 50 mL of a 2.5% (w/v) PEG 4000 solution was prepared; 1 g of JSEC was added to the solution, and the suspension was kept under magnetic stirring at room temperature for 1 h.

The suspension was centrifuged at 4,500 rpm for 10 min, and the resulting solid phase was isolated. The pellet obtained after centrifugation was lyophilized for at least 12 h. Following lyophilization, 80 mL of pure water was added to the capsules, and the suspension was stirred for 4 h. Subsequently, the JSECs were washed three times via vacuum filtration with pure water to remove free PEG weakly adsorbed to the surface. Following the washing step, the samples were lyophilized to dryness and stored for further characterization studies. The final product obtained after this process was named PEG–JSEC.

Starting with an initial amount of 20 g of raw pollen, the final yield of dried PEG–JSEC was approximately 5 g, representing a recovery rate of ~ 25%. This yield level is consistent with the substantial removal of cytoplasmic and lipidic internal components during the sequential chemical treatments. In this process, PEG-4000 acts as a cryoprotectant and a steric stabilizer. It forms a transient physical matrix that mitigates capillary-force-induced collapse and prevents irreversible aggregation of the hollow structures.

### Characterization

The samples were characterized for their structural, morphological, and chemical properties following each purification stage.

#### Scanning electron microscopy (SEM)

The surface morphologies of raw pollen (J), alkali-treated pollen (KJ), and sporopollenin exine capsules (JSEC) were examined using scanning electron microscopy (SEM). Prior to SEM analysis, the samples were mounted onto clean silicon (Si) substrates and sputter-coated with approximately 3 nm of gold to enhance surface conductivity. The coating process was performed using a Quorum Technologies Emitech K550x sputter coater. SEM images were acquired using an FEI Nova NanoSEM 450 instrument under an acceleration voltage of 10.0 kV. Morphological changes and particle sizes of the samples before and after extraction were evaluated based on the obtained SEM images.

#### Brunauer–Emmett–Teller (BET) surface area analysis

The specific surface areas and pore size distributions of the J, KJ, and JSEC samples were determined using a Micromeritics Gemini VII 2390t instrument. The BET surface area was calculated using the linear region of the nitrogen adsorption isotherms (obtained in the P/P₀ = 0–1 range) within the relative pressure range of 0.05–0.30. The pore size distribution was analyzed via the Barrett–Joyner–Halenda (BJH) method using data derived from the adsorption branch. Before measurements, the samples were degassed at 100 °C for 2 h under a nitrogen atmosphere to remove adsorbed moisture and volatile species.

#### FT-IR analysis

The chemical structural properties of the samples were analyzed using a PerkinElmer FT-IR spectrometer in the wavenumber range of 400–4000 cm^− 1^. For FT-IR measurements, the samples were homogenized in an agate mortar for approximately 30 min at a mass ratio of 1% sample to 99% KBr. From the prepared mixture, 200 mg was taken and formed into a pellet; the pressing process was conducted by applying 5 tons of pressure for 5 min, followed by 10 tons of pressure for 10 min. A pure KBr pellet was prepared as a reference.

#### UV–vis spectroscopy

To monitor protein removal throughout the extraction stages, the supernatants obtained after each chemical treatment were analyzed using UV–Vis spectroscopy. Measurements were performed at a wavelength of 280 nm, representing the characteristic absorption of aromatic amino acids in proteins, using a BioTek Epoch instrument with quartz cuvettes. To evaluate protein removal incrementally during the extraction process, supernatants from the following steps were analyzed individually:

- Following the acetone defatting process.
- Following the alkaline treatment with KOH.
- First, second, third, and fourth washings with deionized water after the alkaline treatment.
- Following the acidolysis process with ortho-phosphoric acid.
- First, second, third, and fourth washings with deionized water after the acidolysis treatment.

#### SDS-PAGE analysis

To directly verify protein removal, SDS-PAGE analysis was performed on the J, KJ, and JSEC samples. Protein extraction was performed by incubating the samples in 0.2 M phosphate-buffered saline (PBS, pH 6.8) overnight at 4 °C under continuous agitation. To isolate the soluble protein fraction, the extracts were centrifuged at 16,000 × g for 30 min at 4 °C. The supernatant was subsequently recovered and heated at 95 °C for 5 min in a sample loading buffer containing 2-mercaptoethanol to ensure full denaturation under reducing conditions.

For electrophoretic separation, equal volumes (20 µL) of each sample were loaded into the wells. Sodium dodecyl sulfate-polyacrylamide gel electrophoresis (SDS-PAGE) was executed at a constant voltage of 150 V. Following the run, the gels were fixed in a solution of 50% (v/v) isopropanol and 10% (v/v) acetic acid for 40 min. Protein bands were visualized through incubation in Coomassie Brilliant Blue R-250 (prepared in 10% acetic acid), establishing a detection limit of approximately 10–50 ng of protein bands. After two successive destaining steps to reduce background interference, the molecular weights of the resolved protein bands were calculated using a Broad Multi Color Pre-Stained Protein Standard (GenScript, USA) as the reference scale. A purified *Juglans*-specific allergen standard was not included in the electrophoretic analysis; this absence is acknowledged as methodological limitation regarding the absolute identification of specific residual allergens.

#### Solid-State ¹³C-CP/MAS NMR analysis

Solid-state ¹³C-CP/MAS NMR analyses were performed at room temperature using a JEOL ECZ-500R spectrometer. Samples were packed into 3.2 mm zirconium oxide rotors. Measurements were acquired with a spin rate of 6 kHz, 4000 scans, and a recycle delay of 5 s. Glycine was used as an external standard. The carbon and proton resonance frequencies were set to 125 MHz and 500.13 MHz, respectively.

#### CHNS elemental analysis

The carbon (C), hydrogen (H), and nitrogen (N) contents of the J, KJ, and JSEC samples were determined using a LECO CHNS-932 instrument. The nitrogen-based protein equivalent was calculated by multiplying the measured nitrogen percentage by the Kjeldahl conversion factor (6.25).

## Results and discussion

### SEM analysis of sporopollenin capsule formation from *Juglans regia* pollen

**Fig. 2:**
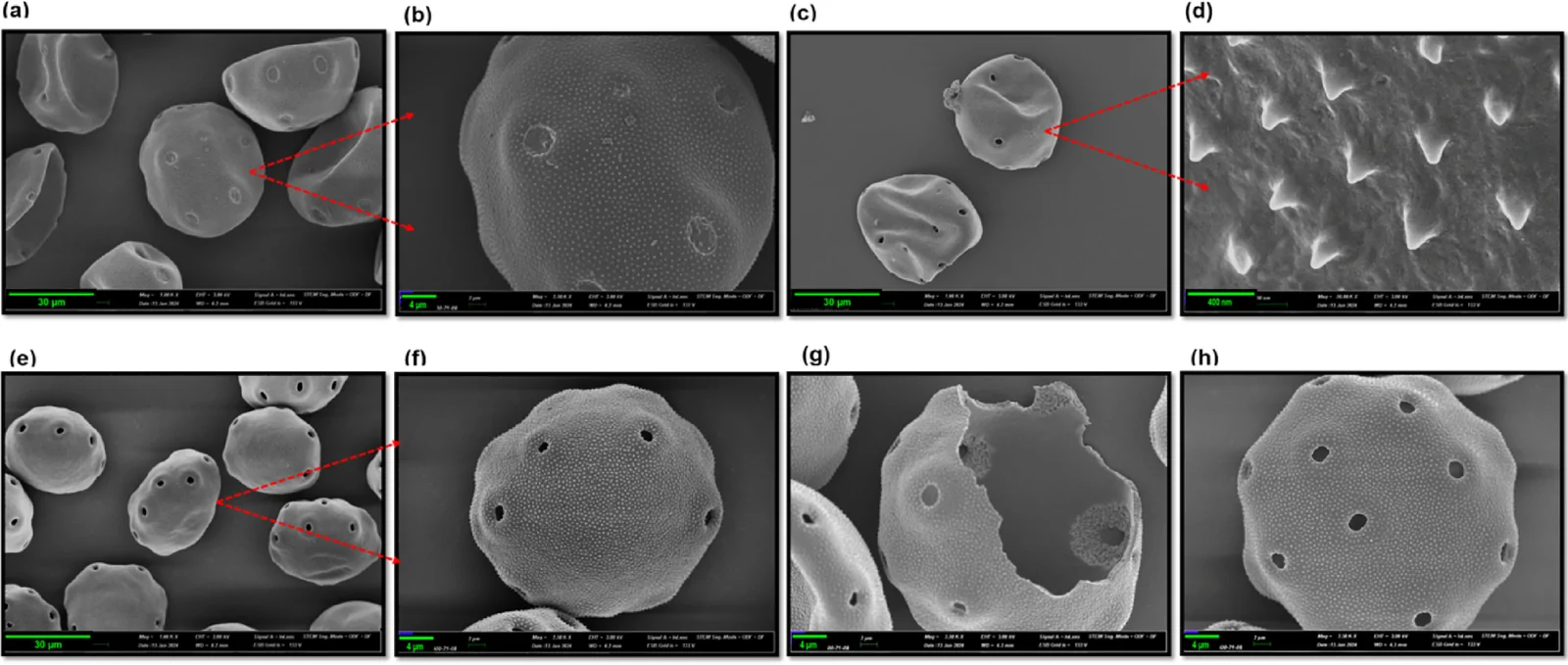
SEM micrographs illustrating the morphological evolution of *Juglans regia* pollen during sporopollenin exine capsule preparation. (**a, b**) Raw pollen grains (J). (**c, d**) Acetone-defatted pollen after lipid removal. (**e, f**) JSEC obtained after sequential KOH and H₃PO₄ treatments. (**g**) Fractured JSEC revealing the internal cavity. (**h**) PEG–JSEC after PEG-4000 stabilization showing improved morphological integrity.

Raw *Juglans regia* pollen (J) exhibited a predominantly spheroidal to slightly oval morphology in SEM images, characterized by a multiporate/pantoporate aperture arrangement and a distinct reticular exine architecture (Fig. 2a-b). The determined pollen sizes, ranging from 38 to 42 μm, and the pore structures are in full agreement with the morphological characterization data reported in the literature^21^. Considering that SEM measurements reflect the widest axis in the image and that the effective size during sieving depends on particle orientation, observing a 38–42 μm range in the fraction passing through a 38 μm sieve is an expected outcome. These morphological parameters served as a fundamental reference for evaluating structural changes following extraction.

In the JSECs obtained through sequential chemical treatments, SEM analysis clearly demonstrated that the internal pollen components (intine and cytoplasmic content) were largely removed, and the sporopollenin-based exine shell became dominant (Fig. 2e-f). At this stage, a partial reduction in pollen size and the increased prominence of surface pores/grooves indicate a morphological transformation consistent with the evacuation of the internal volume. However, limited structural collapse was observed in some capsules. This deformation can be attributed to the reduction in mechanical support provided by the internal content, rendering the thin-walled hollow capsules more susceptible to deformation, particularly under drying or vacuum conditions. Therefore, this condition should not be interpreted as an indicator of unsuccessful extraction but rather as a structural vulnerability that can accompany the formation of hollow capsules.

In the KOH-treated capsules (KJ), an increased tendency toward collapse was observed without a significant change in pollen size. This observation is consistent with the fact that while the alkaline step advances the removal of remaining internal components, it may relatively decrease the mechanical strength of the capsule wall. Although previous studies utilized ultrasonic treatment during the KOH stage^14^, preliminary trials in this study revealed that ultrasonic energy significantly increased the risk of fracturing and cracking in evacuated capsules; therefore, ultrasonic treatment was avoided during the alkaline step. Consequently, while chemical purification was maintained, mechanical stress that could weaken capsule integrity was effectively controlled.

To mitigate the tendency for collapse, JSECs were treated with PEG-4000, followed by lyophilization, and subsequently re-suspended in an aqueous medium with multiple washing steps to remove free PEG. Images of fractured capsules directly confirmed the successful evacuation of the internal volume (Fig. 2g). As observed in the SEM image of PEG-JSEC (Fig. 2h), the chemical treatment facilitated morphological recovery compared to the pristine state. The removal of internal biological support during extraction creates a structural vulnerability, leading to irreversible collapse during lyophilization due to capillary forces. To quantify the recovery, a manual count of *n* = 150 particles was performed across three representative regions. The results (summarized in Supplementary Table S1) show that 83.3% of the capsules successfully maintained their spherical geometry, while only 4.7% exhibited minor clustering, confirming the role of PEG-4000 as a steric stabilizer.

These findings reveal that PEG-4000 treatment provides a “structural recovery” effect that reduces irreversible drying-induced collapse and deformation, significantly improving the morphological integrity of the final product. Overall, the SEM results clearly demonstrate the successful acquisition of hollow sporopollenin exine capsules from *Juglans regia* pollen and highlight the critical role of PEG-4000 application in stabilizing capsule morphology.

### BET surface area and pore structure

**Fig. 3:**
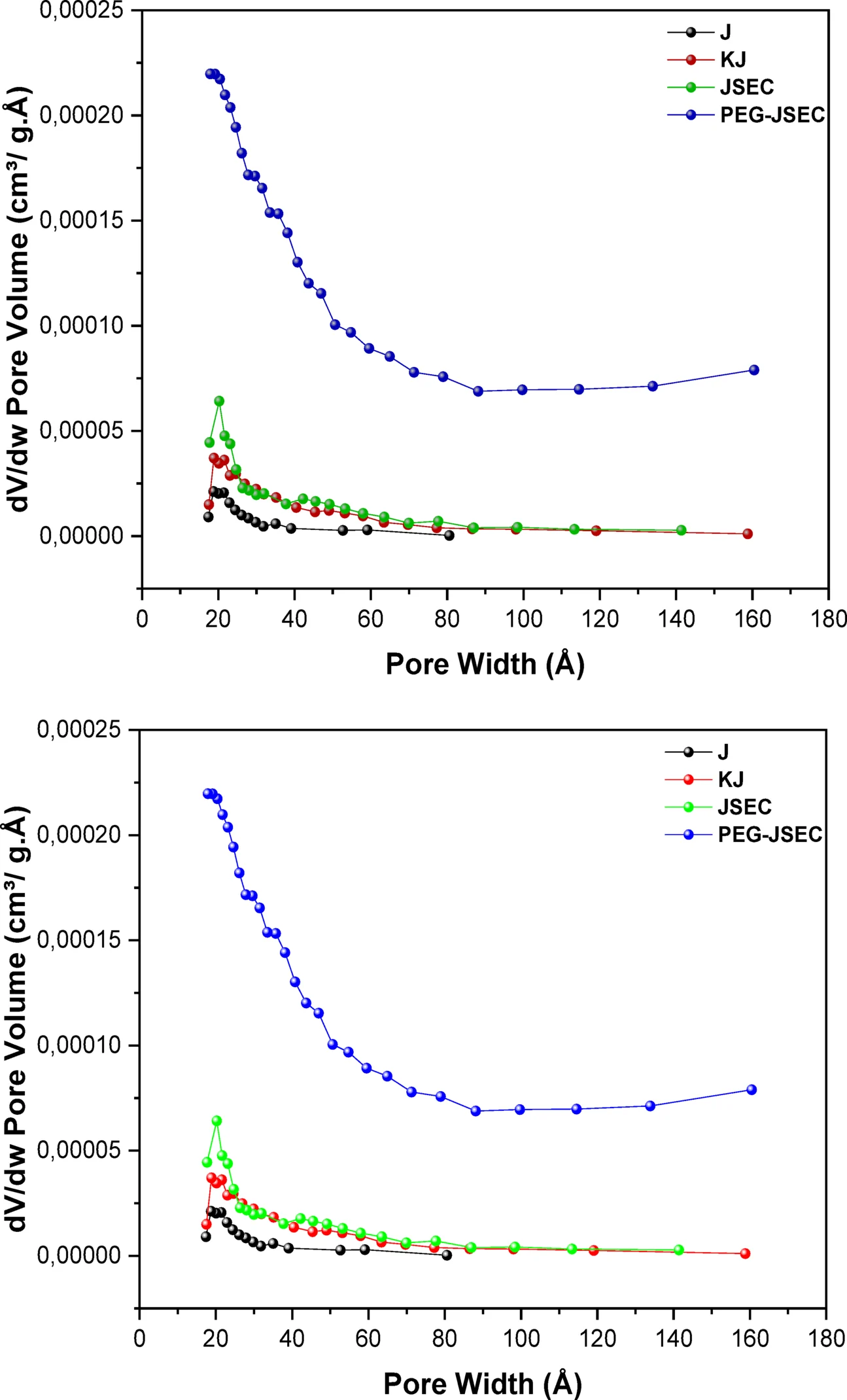
(**a**) Nitrogen adsorption–desorption isotherms and (**b**) pore size distributions calculated by the BJH method for raw *Juglans regia* pollen (J), sample obtained after acetone defatting and KOH treatment (KJ), sporopollenin exine capsules obtained after H₃PO₄ acidolysis (JSEC), and PEG-assisted stabilized capsules (PEG–JSEC).

Figure 3 presents the nitrogen adsorption–desorption isotherms and the pore size distributions calculated using the BJH method for *Juglans regia* pollen subjected to sequential chemical treatments (Table 1). Untreated *Juglans regia* pollen exhibited a limited adsorption capacity with a low BET surface area of 1.10 m^2^/g and a pore volume of 0.339 × 10⁻³ cm³/g. This situation arises from the restricted access of nitrogen molecules to the structure due to the intine layer and cytoplasmic content largely filling the pores.

**Table 1:**
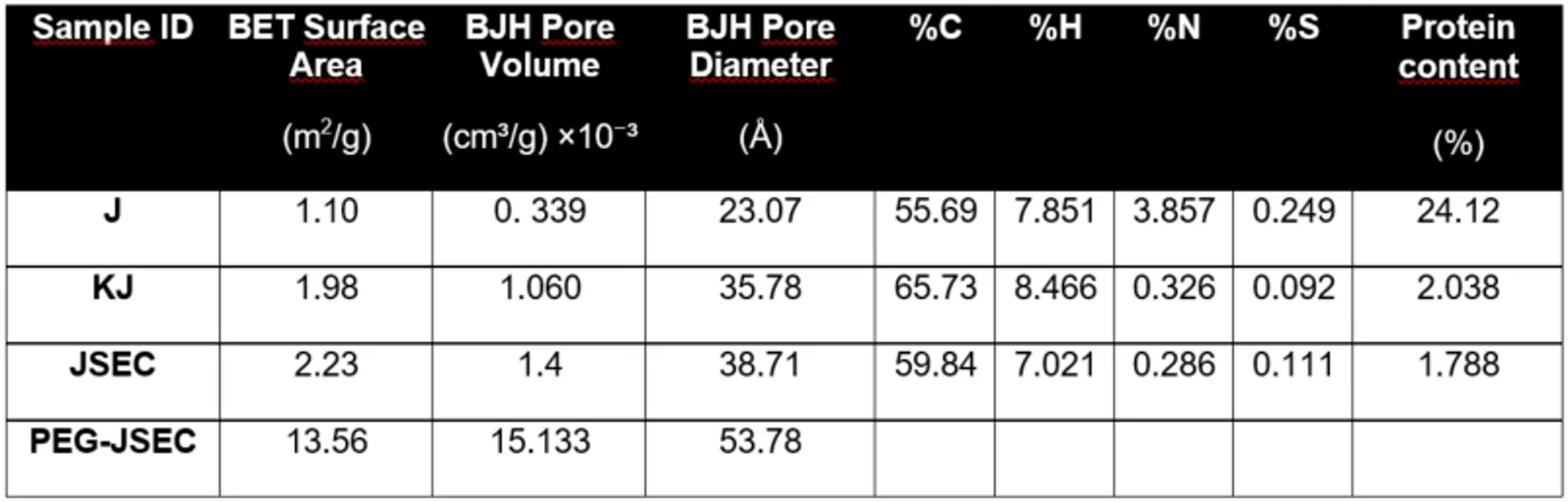
Changes in surface parameters and elemental composition of *Juglans regia* pollen following sequential chemical treatments.

In the KJ sample obtained after acetone defatting and KOH treatment, it is observed that the BET surface area increased to 1.98 m^2^/g and the pore volume reached 1.060 × 10⁻³ cm³/g. This increase can be explained by the removal of lipids and the denaturation and removal of proteins during the alkaline treatment, which rendered the sporopollenin exine more accessible. However, it is understood that the capsule structure was largely preserved at this stage, and thus the increase in surface area remained limited.

In the JSECs obtained after acid treatment, the surface area increased to 2.23 m^2^/g, the pore volume to 1.40 × 10⁻³ cm³/g and the average pore diameter to 38.71 Å. These results indicate that the acidolysis step performed with H_3_PO_4_ ensured the removal of remaining protein and intine residues, making the internal cavity of the capsule more prominent and partially expanding the pores. Conversely, it is understood that the capsule integrity was largely maintained due to the absence of ultrasonic treatment.

The PEG-JSEC sample, obtained after PEG-supported processing and subsequent lyophilization, exhibited the highest BET surface area (13.56 m^2^/g) and pore volume (15.13 × 10⁻³ cm³/g) values. This significant enhancement in surface area is structurally consistent with the prevention of macroscopic collapse, as evidenced by the high morphological integrity observed in wide-field fluorescence microscopy (Supplementary Fig. S1). Furthermore, the preservation of the open-pore network is directly visualized in the SEM micrographs (Fig. 2h). The reduction in inter-particle aggregation via steric stabilization ensures maximum accessibility to both the internal cavity and the reticulated exine pores, preventing the surface area loss typically associated with structural collapse during drying. This total accessible surface area, which includes both the external wall and the internal cavity accessed via the reticulated exine channels, is notably higher than the typical values reported for non-stabilized Lycopodium clavatum SECs (approx. 1–3 m²/g). This elevation validates the effectiveness of the PEG-4000 stabilization, which successfully prevented the capillary-induced collapse of the capsules during drying. Furthermore, functionality and pore accessibility are quantitatively confirmed by the BJH pore size distribution analysis derived from the N₂ desorption branch (Fig. 3). The PEG-JSEC structures exhibit an accessible mesoporous network with an average pore width of 5.38 nm (53.78 Å) (Fig. 3b), demonstrating excellent permeability for potential loading applications. The average pore diameter reaching 53.78 Å reveals that PEG acted as a temporary stabilizer during the drying process, successfully preventing capsule collapse and contributing to the formation of a more open and homogeneous pore network. PEG’s capacity to prevent pore collapse by forming a crystalline or amorphous matrix during the freeze-drying stage is consistent with similar polymeric stabilization mechanisms in the literature^22^.

When the pore size distribution curves are examined, it is seen that the untreated pollen has a narrow pore distribution, while this distribution expands toward the mesopore region in the KJ and JSEC samples. In the PEG-JSEC sample, a significant increase in pore volume was observed particularly in the 20–150 Å range, indicating the formation of a structure more favorable for adsorption and molecular diffusion.

Consequently, BET and BJH analyses clearly demonstrate that the sequential chemical treatments applied to *Juglans regia* pollen gradually improved the surface area and pore structure. The transition from raw pollen to the JSEC structure obtained after KOH and acid treatment provided a controlled pore opening, while the PEG-supported process enabled the production of microcapsules with higher porosity and structural stability. These findings indicate that the obtained sporopollenin capsules offer a suitable structure for advanced surface characterizations and application-oriented studies.

The structural parameters of *Juglans regia*-derived capsules offer distinct advantages compared to *Lycopodium clavatum* (typically ~ 25 μm), which is widely studied in the literature. The larger particle size of *Juglans regia* pollen (38–42 μm) theoretically provides a larger internal cavity volume, which is of critical importance for high-capacity drug loading applications. Furthermore, the significant increase in BET surface area to 13.56 m^2^/g following the PEG stabilization and subsequent lyophilization process indicates that Juglans SECs possess a highly accessible pore network. The fundamental role of PEG in this process is to provide structural stability by preventing the collapse of the pore structure during the freeze-drying stage. The removal of PEG through the water washing process performed after lyophilization left behind a clean and open pore pathway. During this process, PEG-4000 acts as a cryoprotectant during lyophilization. It forms a transient matrix that provides steric stabilization, effectively preventing the capillary-force-induced collapse of the hollow capsule structure^22^. The high surface area and structural integrity of the obtained Juglans SECs more than meet the morphological standards required for a wide range of applications, extending from the oral delivery of chemotherapeutic agents^23^ to the development of smart hybrid systems containing nanozymes^9^. Such morphological improvements are a fundamental requirement for optimizing drug encapsulation efficiency and release kinetics^6^. Additionally, these superior structural properties make these capsules advanced candidates for complex molecular separation processes. For example, considering that the selective absorption of ssDNA has been achieved using smaller pollen templates in the literature^4^, the higher internal volume and enhanced porosity offered by our JSECs have the potential to provide higher loading capacity in specialized fields such as genetic material isolation. Furthermore, the extraordinary chemical and structural stability of this material has allowed it to be successfully integrated into advanced technological applications, such as supercapacitor electrode design^12^. Unlike commercially sourced alternatives, *Juglans regia* pollen offers a significant advantage as a sustainable biomass waste, due to its local accessibility, cost-effectiveness, and long-term supply capacity.

### FT-IR analysis results

**Fig. 4:**
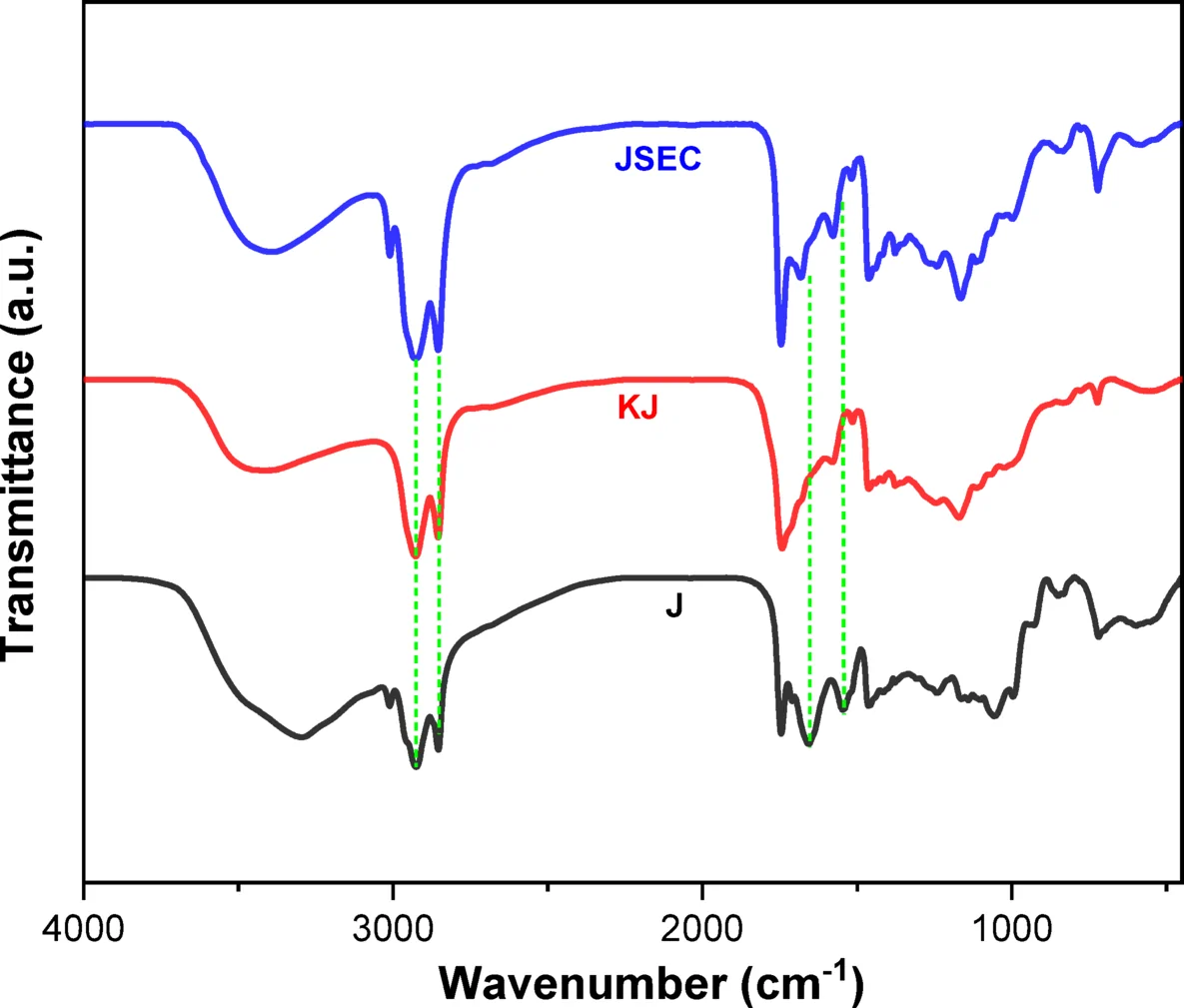
FT-IR spectra of raw *Juglans regia* pollen (J), alkali-treated sample (KJ), and sporopollenin exine capsules (JSEC).

The FT-IR spectra of *Juglans* samples demonstrate that as the chemical purification stages progress, the protein components of the pollen content decrease, and the sporopollenin-based exine structure becomes dominant (Fig. 4). Since all KBr pellets were prepared with an identical sample mass, the variations in band intensity directly reflect the underlying compositional transformations.

The significant attenuation of the Amide I (≈ 1650–1660 cm⁻¹) and Amide II (≈ 1540 cm⁻¹) bands, which are prominent in the raw pollen sample, during the alkaline treatment phase indicates that this stage effectively contributes to the removal of protein/peptide-derived components. The reduction of these bands to minimal levels in the JSEC sample following sequential treatments molecularly confirms the success of the deproteinization process. This is a critical prerequisite for sporopollenin micro-carriers to exhibit a biocompatible and safe profile in potential for preclinical development^9^.

The variations in the C–H stretching bands (2800–3000 cm⁻¹) associated with aliphatic components throughout the processing steps are consistent with the reduction of lipophilic components and/or the relative compositional shifts within the exine matrix. Conversely, the lack of substantial disappearance of the broad O–H band in the 3300–3400 cm⁻¹ range demonstrates that the purification process, while targeting internal components, did not entirely eliminate the essential oxygenated functional groups of the exine wall.

This preservation of functional groups proves that the resulting capsules maintain the chemical activity necessary for drug loading capacity and surface interactions^23^. Consequently, the FT-IR results confirm that the *Juglans* pollen has been both cleared of allergenic proteins and its structural functionality has been preserved without degradation.

### UV–vis spectroscopy: Evidence of protein removal

**Fig. 5:**
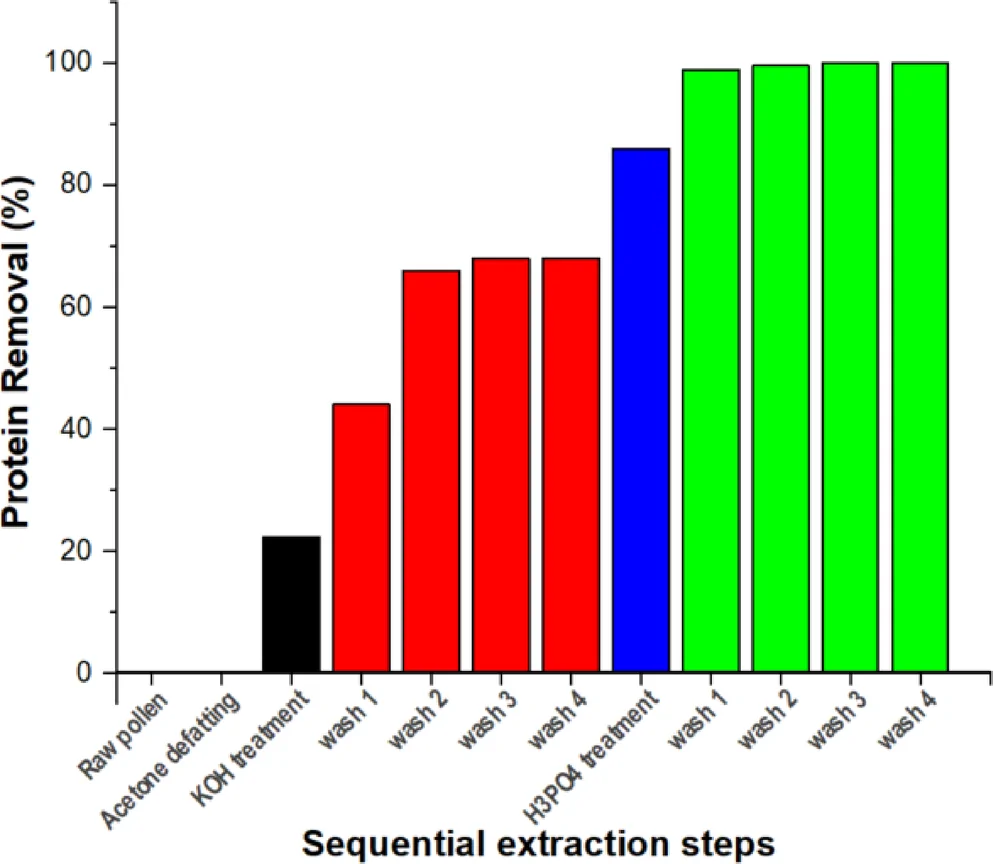
Protein removal efficiency (%) as a function of sequential SEC extraction steps during the preparation of *Juglans regia* sporopollenin exine capsules.

UV–Vis analyses demonstrate that protein removal during the synthesis of JSEC from *Juglans regia* pollen occurs gradually and irreversibly throughout the extraction stages (Fig. 5). The limited extent of protein removal following the acetone defatting process indicates that this step primarily serves to eliminate lipophilic components, while proteins remain largely retained within the structure.

Figure 5 Protein removal efficiency (%) as a function of sequential SEC extraction steps during the preparation of *Juglans regia* sporopollenin exine capsules.

In contrast, a significant increase in protein removal was observed following alkali (KOH) treatment. This increase is attributed to the denaturation of proteins in the alkaline medium and their subsequent transition into the solution phase, signifying that the protein removal process effectively initiates at this stage. The gradual increase in protein removal during the subsequent distilled water washes indicates that dissolved proteins are largely eliminated during the initial washing steps, with successive washes serving a complementary role to complete the process.

The acidolysis step, performed with ortho-phosphoric acid (H₃PO₄), emerges as the critical stage where protein removal reaches its maximum level. It was determined that protein removal exceeded 95% following this treatment, with only limited changes observed in the subsequent washing steps. This finding clearly demonstrates that the acidolysis step possesses high efficiency in eliminating residual proteins and protein fragments, marking the completion of the deproteinization process at this stage.

### SDS-PAGE results

According to the WHO/IUIS Allergen Nomenclature Sub-Committee (Allergen.org), no purified allergens have been officially characterized for *Juglans* pollen to date. However, comparative analyses indicate that the primary allergen of *Betula*, a genus belonging to the same order as *Juglans*, exhibits Pathogenesis-Related (PR) protein activity analogous to Jug r 5 (20 kDa) identified in *Juglans regia*. Furthermore, the minor allergen from *Betula pendula* is classified as a profilin, showing structural similarities to Jug r 7 (13 kDa).

**Fig. 6:**
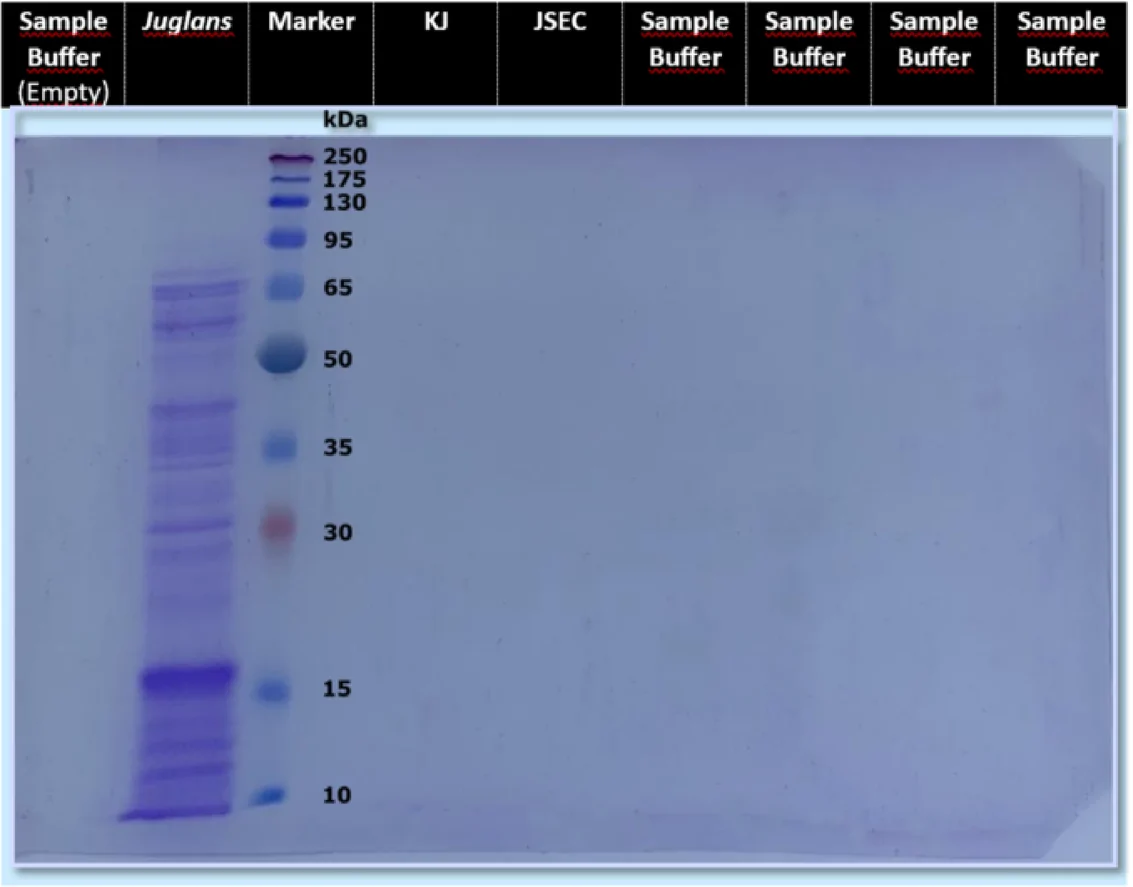
SDS-PAGE profiles of raw *Juglans regia* pollen (J) and chemically treated samples (KJ and JSEC), demonstrating effective protein removal after sequential extraction steps. The detection threshold for SDS-PAGE is ~ 10–50 ng per band.

While these specific allergenic protein fractions and a faint smearing pattern above 20 kDa, indicative of partial protein degradation by endogenous pollen proteases during the overnight aqueous extraction, were observed in the raw *Juglans* pollen extract (J), no corresponding protein bands or residual smears were observed in the analyzed processed pollen samples, specifically KJ and JSEC (Fig. 6). The absence of detectable bands in KJ and JSEC lanes, below the Coomassie-based detection threshold of ~ 10–50 ng per band, confirms removal of allergenic proteins to sub-detectable levels. This complete molecular clearance, evidenced by the lack of any residual fragments, confirms that the chemical treatment protocols successfully reduced the concentrations of both PR-proteins and profilins to levels below the detection limits of SDS-PAGE, ensuring the deproteinization required for the development of hollow microcapsules.

### Solid-State ¹³C-CP/MAS NMR analysis results

**Fig. 7:**
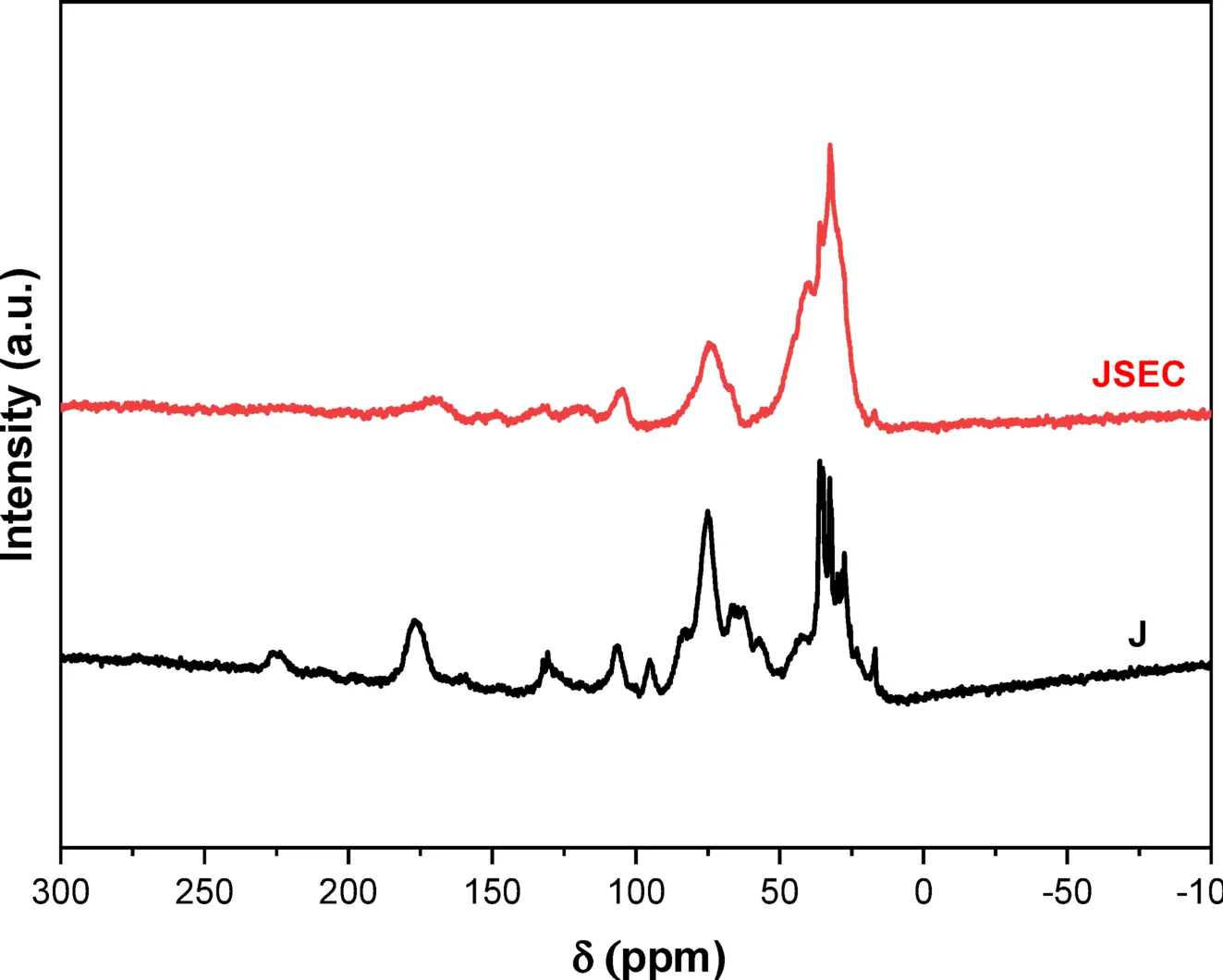
Solid-state ¹³C-CP/MAS NMR spectra of raw *Juglans regia* pollen (J) and protein/lipid-removed exine microcapsules (JSEC).

A comparison of the ¹³C-CP/MAS NMR spectra of raw *Juglans regia* pollen (J) and the JSEC sample subjected to protein/lipid removal reveals a significant reduction in protein-associated signal contributions post-extraction (Fig. 7). Specifically, the relative decrease observed in the carbonyl region (amide/ester C = O) within the 160–180 ppm range in JSEC is consistent with the removal of protein/peptide-derived components. Similarly, the attenuation of carbon environments attributable to the protein backbone in the 50–65 ppm range supports the substantial elimination of protein content. It is important to acknowledge that native sporopollenin inherently contains aliphatic carbons (e.g., from fatty acid chains) that resonate broadly in the 50–65 ppm region^24^. Therefore, the attenuation of the relatively sharper signals confirms the successful removal of amino acid α-carbons, while the remaining broad background signal in this region is attributed to the intrinsic aliphatic network of the native sporopollenin exine. Furthermore, while the harsh acidolysis conditions (85% H₃PO₄ at 70 °C) carry a potential risk of partial sporopollenin degradation via ester hydrolysis, the structural integrity of the final JSEC framework was successfully preserved. This preservation is strongly supported by the retention of these characteristic sporopollenin signatures in the NMR and FT-IR spectra, as well as the intact capsular morphologies observed in the SEM micrographs. This high level of purity, evidenced by NMR data, is in full alignment with the requirement highlighted by Mundargi et al.^23^ that orally administered carriers must not trigger an immune response. This marked minimization of protein signals molecularly validates the fundamental criteria for developing the biocompatible and clinically safe ‘smart’ drug delivery platforms defined by^9^. Additionally, the shift in signal distribution within the 0–50 ppm aliphatic region and the 110–160 ppm aromatic/olefinic region in JSEC indicates that the spectral profile of the sample post-purification aligns more closely with the exine/sporopollenin character. Furthermore, the persistence of O-alkyl carbon signals in the 60–110 ppm range suggests that oxygenated carbon environments have not been entirely eliminated.

To rigorously distinguish protein-derived alpha-carbons from intrinsic oxygenated aliphatic structures, a detailed spectral deconvolution of the 52–85 ppm region was performed (Supplementary Fig. S2). This resolution confirmed that while the proteinaceous signal at ~ 56 ppm is significantly attenuated post-purification, the signal at ~ 64 ppm, which is attributed to secondary alcohols (C-OH) in the polyvinyl alcohol-like units of the sporopollenin polymer^25^, remains persistent. This mathematical evidence substantiates that the residual intensity in this region is a characteristic feature of the native sporopollenin framework rather than residual protein.

### CHNS elemental analysis and estimated protein content

The results of the CHNS elemental analysis are summarized in Table 1. The raw pollen sample (J) exhibited high nitrogen content, with the calculated protein content determined to be 24.12%. Following the alkali treatment (KJ), the protein content significantly decreased to 2.04%. In the JSEC sample, obtained after consecutive processing steps, this value further declined to 1.79%, demonstrating that protein-based components were extensively removed. The residual nitrogen content (1.79%) is primarily attributed to the intrinsic nitrogen of the sporopollenin framework; however, contributions from Maillard-type adducts or products of incomplete hydrolysis during intensive chemical processing cannot be entirely ruled out.

The sulfur content also showed a marked reduction compared to the raw pollen and remained at minimal levels. These findings confirm that the chemical purification stages effectively eliminated the internal constituents of the pollen, successfully yielding hollow sporopollenin exine capsules.

## Study limitations

Although this study successfully establishes the structural and chemical verification of JSECs, the absence of direct allergenicity assays (e.g., IgE ELISA) and in vivo biocompatibility data limits the immediate clinical assessment. Furthermore, while SEM provided the foundational size range and wide-field fluorescence microscopy quantitatively validated the morphological recovery, broader size distribution profiling and comprehensive assessments of batch-to-batch reproducibility have not yet been evaluated. However, these aspects are being addressed in our ongoing research project (TÜBİTAK 223M188) to fully validate the platform’s potential for preclinical development. Although PEG was removed by aqueous washing, trace residues cannot be entirely excluded without complementary analysis (e.g., TGA or XPS). This is acknowledged as a minor uncertainty in surface composition.

## Conclusion

The present study successfully demonstrates the isolation of high-purity sporopollenin exine capsules (JSECs) from *Juglans regia* pollen while maintaining their unique hierarchical morphology. The integration of solid-state ¹³C-CP/MAS NMR, SDS-PAGE, and CHNS analysis provided a comprehensive molecular-level verification of protein removal, ensuring that allergenic fractions were eliminated below detectable thresholds, as assessed by SDS-PAGE (detection limit ~ 10–50 ng), solid-state NMR, and FT-IR. The absence of detectable bands in KJ and JSEC lanes, below the Coomassie-based detection threshold of ~ 10–50 ng per band, confirms removal of allergenic proteins to sub-detectable levels. This systematic purification achieving a protein reduction from 24.12% to 1.79% with a 25% yield confirms the high efficiency and reproducibility of the optimized protocol.

A significant contribution of this work is the resolution of the structural collapse issue through a PEG-4000-mediated cryoprotectant-based strategy, where PEG acts as a cryoprotectant to prevent capillary-force-induced collapse during lyophilization, which successfully preserved the capsules’ spherical architecture. The resulting 12-fold increase in BET surface area (from 1.10 to 13.56 m^2^/g) underscores the superior textural stability and pore accessibility of JSECs compared to raw pollen.

In conclusion, *Juglans regia* emerges as a sustainable, locally accessible, and high-performance biomass source for natural microcarriers. The superior internal volume and open pore network of JSECs provide a robust platform for advanced biomedical and biotechnological applications, such as smart drug delivery and hybrid nanozyme systems. This study establishes the fundamental morphological and safety standards required for the potential for preclinical development of sporopollenin-based microtechnologies. Comprehensive in vitro and in vivo biological evaluations of these structures are currently ongoing as part of a national research project (TUBITAK 223M188) and will be reported in future studies.

## Supplementary Information

Below is the link to the electronic supplementary material.


Supplementary Material 1


## Data Availability

The datasets generated during and/or analyzed during the current study are available from the corresponding author on reasonable request.
