# Supplementary material for: Development of morphologically restored and deproteinized Juglans regia pollen-derived sporopollenin exine capsules: A multi-analytical verification study
Source: Sci Rep. 2026 May 22;16:27059. doi: 10.1038/s41598-026-53033-1 (PMC13522523; doi:10.1038/s41598-026-53033-1)
Supplement: Supplementary file 1 [file 41598_2026_53033_MOESM1_ESM.docx]

**Supporting Information**

**Development of Morphologically Restored and and Deproteinized *Juglans regia* Pollen-Derived Sporopollenin Exine Capsules: A Multi-Analytical Verification Study**

Funda Ersoy Atalay^1,*^_,_ Seher Guler^1^, Harun Kaya^2^, Senol Alan^3^

^1^Inonu University, The Faculty of Science and Arts, Department of Physics, Malatya, Türkiye

^2^Malatya Turgut Ozal University, The Faculty of Engineering and Natural Sciences, Malatya, Türkiye

^3^Zonguldak Bulent Ecevit University, Faculty of Science, Department of Biology, Zonguldak, Türkiye

^*^Corresponding author: funda.atalay@inonu.edu.tr

https://orcid.org/0000-0002-5776-2490

Number of pages: 3; Number of figures:2; Number of tables1

**a)**

**b)**

**d)**

**c)**

**Figure S1.** Comparative fluorescence microscopy of (a, b) JSEC and (c, d) PEGSEC at (a, c) 10x and (b, d) 40x. Quantitative analysis (n=150) confirms 83.3% spherical recovery in PEG-stabilized capsules. The results demonstrate that PEG-4000 provides steric stabilization, preventing irreversible aggregation and structural collapse. This morphological preservation directly correlates with the 12-fold increase in BET surface area from 1.10 to 13.56m^2^/g.

**Table S1.** Statistical Morphological Analysis of PEG-JSEC. A quantitative assessment of n=150 capsules was performed across three independent wide-field fluorescence microscopy regions .

| Morphological Status | Count (n=150) | Percentage (%) |
| --- | --- | --- |
| Spherical and Intact | 125 | 83.3% |
| Partially Collapsed | 18 | 12.0% |
| Aggregated | 7 | 4.7% |

**Figure S2.** Detailed deconvolution of the ¹³C-CP/MAS NMR spectra in the 52–85 ppm region for *Juglans* *regia* and JSEC samples. The overlapping signals were resolved into distinct Gaussian components to differentiate protein-derived α-carbons from native oxygenated aliphatic structures.
